# Trends and Disparities in the Prevalence of Childhood Obesity in South Texas between 2009 and 2015

**DOI:** 10.1155/2017/1424968

**Published:** 2017-07-18

**Authors:** Byron A. Foster, Trevor M. Maness, Christian A. Aquino

**Affiliations:** ^1^Department of Pediatrics, University of Texas Health Science Center at San Antonio, San Antonio, TX, USA; ^2^School of Public Health, University of Texas Health Science Center at Houston, Houston, TX, USA

## Abstract

**Background:**

Recent reports have highlighted possible decreases over time in obesity, particularly among children aged 2–5 years. Hispanic children experience significantly higher obesity rates, and less is known about trends for Hispanic children.

**Methods:**

A large healthcare system-based dataset from south Texas was used to analyze body mass index (BMI) values obtained clinically from 2009 through 2015. Age and race/ethnicity specific prevalence of overweight and obesity were calculated using CDC standards and trends were examined over time using regression analyses, and mapping software was used to identify geographic variation.

**Results:**

Hispanic children in south Texas experience levels of obesity (25.3%, 95% CI: 25.1–25.6) significantly higher than their white (16.6%, 95% CI: 16.0–17.2) or black (18.2%, 95% CI: 17.3–19.1) peers. Obesity in Hispanic children aged 2–5 years decreased from 18.5%, 95% CI: 16.6–20.5, in 2009 to 15.1%, 95% CI: 14.3–15.9, in 2015. Obesity in Hispanic adolescents was stable at 30.4%, 95% CI: 28.5–32.4, in 2009 and 31.3, 95% CI: 30.3–32.2, in 2015.

**Conclusions:**

While obesity decreased in the youngest age group of Hispanic children, south Texas continues to experience high levels of obesity that exceed national averages with significant disparities.

## 1. Introduction

Multiple analyses of nationally representative data show an overall increase in obesity from the 1980s through the 1990s followed by a more recent plateau with no significant increase in the prevalence of obesity since the late 2000s [1–3]. These data also suggest a decrease in the prevalence of obesity for children aged 2–5 years [1, 2]. This decrease is primarily in white children while Hispanic children continue to experience a higher rate of obesity [1]. This disparity is particularly concerning given our understanding of weight trajectories [4] and the higher risk of both liver disease and diabetes seen in the Hispanic population [5]. For children aged 6–11 years, national data showed a recent plateau and, for adolescents, a continued increase in the prevalence of obesity across racial and ethnic groups [1].

A large, healthcare-based cohort in southern California showed a significant reduction in the prevalence of overweight, obesity, and extreme obesity from 2008 to 2013, in both the white and Hispanic populations [6]. Also, a recent study from Ohio using an academic medical center's records showed a similar plateau in overweight and obesity prevalence from 2011 to 2014 [7]. These data from individual localities and regions are valuable in both substantiating national trends and identifying important differences that may help identify policy-level effects or environmental changes driving regional differences. For example, a recent report from the Centers for Disease Control and Prevention examining participants in the Special Supplemental Nutrition Program for Women, Infants, and Children (WIC) identified important differences in prevalence between Los Angeles and New York [8], and another report using WIC data from Oklahoma showed no decline in obesity prevalence from 2005 to 2010 [9].

San Antonio, Texas, is the seventh largest city in the United States with 55% of the population being Hispanic making it an important potential indicator of obesity trends for Hispanic children. Data from the local Health District indicate that 65% of the adult population are overweight or obese, and 30% of the high school students population are overweight or obese [10].

Here we describe trends in obesity prevalence for children aged 2–17 years using a healthcare dataset. The health system is one of the three largest providers of children's healthcare in San Antonio.

## 2. Methods

### 2.1. Data Source

A dataset from a healthcare system (University Health System) in San Antonio, Texas, was used to generate the estimates. Height and weight are measured during clinic or hospital encounters by clinical staff. Race and ethnicity categories are based on self-reported data.

Biologically implausible values were removed from the dataset (BMI *z*-scores < −5 and >8). From 2009 to 2015, there were 114,406 individuals with 352,744 visits for children between 2 and 17 years of age with height and weight entered. Individuals had between 1 and 110 visits with a biologically plausible BMI in the record over the seven years of data with a mean of 2.4 and a median of 2 measurements per year (Supplemental Table 1, in Supplementary Material available online at https://doi.org/10.1155/2017/1424968).

### 2.2. Statistical Analysis

EpiInfo (Centers for Disease Control) was used to generate BMI *z*-scores using the CDC 2000 criteria. BMI *z*-scores were categorized for this analysis as normal weight: >−2 through 1.03; overweight: ≥1.04 (equivalent to ≥85th percentile); obese: ≥1.65 (equivalent to ≥95th percentile) [11].

Each individual is represented once per year. The median of each individual's BMI *z*-scores for the year was calculated and used to categorize that individual in that year to minimize the effect of any outlying measurements.

Absolute changes over time were calculated using the absolute difference between estimates and relative changes over time calculated using the absolute change divided by the baseline estimate (2009 data). Generalized linear models were used to account for an individual child occurring in more than one year with thus repeated measures of their weight status. We examined trends over time with the binary dependent outcome of obesity and calendar year, sex and race/ethnicity included as independent variables. SPSS 23.0 (IBM, USA) was used for analyses.

Maps were created using Tableau (version 10.0) and individuals were only counted once per year. Zip codes having less than 10 observations in a year were censored (excluded from the map data).

### 2.3. Ethics

The Institutional Review Board for the University of Texas Health Science Center at San Antonio approved the data collection.

## 3. Results

There were 352,744 data points evaluated representing 114,406 individual children (see Supplemental Table 1) in the final sample with measurements from January 2009 through December 2015 with 50.3% being male and 78.9% of Hispanic ethnicity. Overall, 16.2% (95% CI: 16.0–16.5) of children were overweight and 23.4% (95% CI: 23.2–23.7) of children were obese. Hispanic children had the highest proportion of overweight or obesity compared with other racial and ethnic groups (Tables 1 and 2).

**Figure 1:**
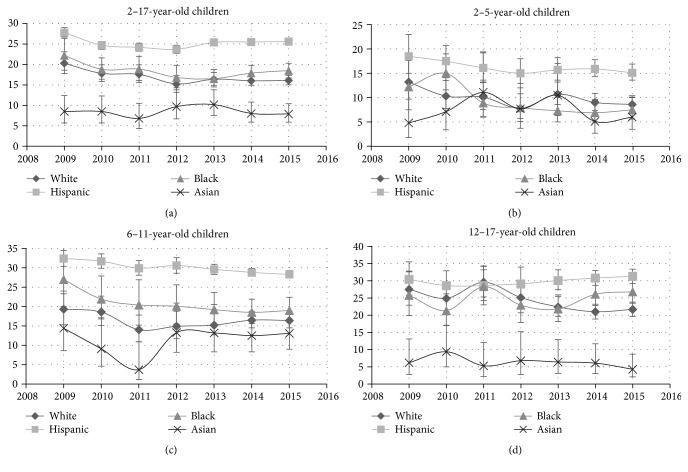
Prevalence of obesity in children in San Antonio over time, 2009 through 2015, with point estimates of percent of obese population shown with 95% CI as error bars. (a) All age groups; (b) 2–5-year-old children; (c) 6–11-year-old children; (d) 12–17-year-old children.

Examining trends over time among Hispanic children (Figure 1, Table 3), overall there was an absolute reduction in obesity of 2.2% (relative reduction of 7.9%) from 2009 to 2015. In the multivariable model including time, Hispanic children had an odds ratio of 1.71 (95% CI: 1.61–1.82) compared with white children of being obese (Table 2). For 2–5-year-olds, an absolute reduction of 3.4% (relative reduction of 18.4%) was observed between 2009 and 2015, with every year starting in 2011 showing a significant reduction compared with 2009 (Table 3). In contrast, there was no difference over time for the 12–17-year-old age group (*p* > 0.05), for all years examined (Table 3). There was a significant decrease seen over time in the unadjusted model for 6–11-year-old Hispanic children that was not sustained after accounting for sex in the final model (Table 3).

White children experienced overall reductions in obesity from 20.3% in 2009 to 16.1% in 2015 for an absolute reduction of 4.2% (relative reduction of 20.7%) (Figure 1, Table 3). Similar to the findings for Hispanic children, the most consistent decrease comparing years was in the 2–5-year-old age group (Table 3).

For black children in this sample, a similar reduction in obesity in the 2–5-year-old population was observed though the differences between baseline (2009) and subsequent years were less consistent than in the Hispanic group with only 2013 and 2014 having a significant reduction compared with 2009 (Table 3). The changes in other age groups of black children were not significant. Notably, black 12–17-year-olds were the only subgroup where females were more likely to be obese than males (OR = 1.30 (95% CI: 1.04–1.62)). No significant trends for Asian children in obesity were found.

**Figure 2:**
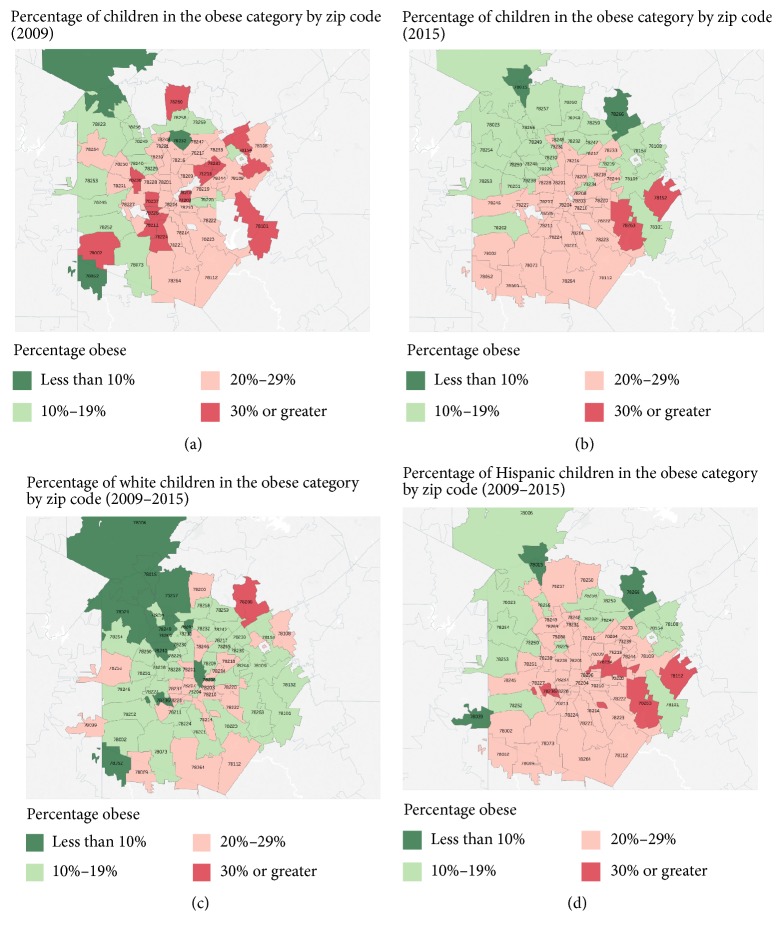
Percentage of children with obesity showing (a) 2009 and (b) 2015 distributions over San Antonio by postal zip code and (c) percentage of white children with obesity from all years (2009–2015) and (d) percentage of Hispanic children with obesity from all years (2009–2015). Dark green indicates less than 10% obese, light green 10–19% obese, light red 20–29% obese, and dark red 30% or greater obese in the indicated zip code.* Note.* zip codes with less than 10 observations were censored (in all figures).

Figure 2 shows a graphical representation of the proportion of children by zip code who are obese. Comparing 2009 (panel (a)) to 2015 (panel (b)), there are fewer zip codes with 30% or greater children who were obese in 2015 compared with 2009 with significant heterogeneity across the mapped region (Figure 2). Panels (c) and (d) show the distribution of white and Hispanic obese children, respectively, with striking differences in the prevalence of obesity by ethnicity across the San Antonio metropolitan area.

## 4. Discussion

In south Texas where adult obesity rates are higher than the national average [12], there have been limited data on the prevalence and trends over time in Texas for children. The data presented here show a higher prevalence of obesity overall (23.4%, 95% CI: 23.2–23.7) compared with both recent national estimates (17.0%, 95% CI: 15.5–18.6) [2] and an estimate from a healthcare-based dataset from southern California (17.5%) [6]. The overall prevalence of obesity is also higher than the overall prevalence in the study from Ohio (19.5%) that also used a healthcare dataset, though the prevalence among Hispanics was 26.3% in that study, and the relatively small proportion of Hispanics in the population explains that difference [7]. Data from the WIC population which has a lower income and it is more similar to our population in socioeconomic status have a more similar estimate of 14.9% for obesity in Texas in 2014 for children aged 2–4 years [13].

The finding of a decrease among children aged 2–5 years is consistent with recent national reports [2]. Two different groups examining Massachusetts school-based data [14] and healthcare-based data similar to this dataset [15] found a decline in obesity among the youngest children [14, 15]. One notable outlier is the Ohio report which found a significant recent increase for 2–5-year-old girls [7]. The decreases in the youngest children are encouraging given the strong correlation between weight at that age and weight status in both adolescence and early adulthood [4, 16, 17].

In San Antonio, there have been multiple recent efforts to address obesity. The local health department with the city led an effort to improve the built environment and facilitate physical activity programs. Additionally, Head Start and PreK4SA, programs for early childhood education, have focused their efforts on making their menus healthier and promoting physical activity at a younger age, and recent studies have found that Head Start participation can lead to a healthier weight [18]. Most of the efforts outlined above are focused on prevention of obesity; however, there are limited resources for treating obesity in children in San Antonio. This may explain why we see a decrease in the youngest age group while also observing a potential increase or at least no change in obesity for adolescents, working under the rubric that more intensive therapies are required for treatment of obesity [19–21]. The disparities seen by race/ethnicity are striking with Hispanic children at much higher risk of obesity compared with their white peers.

For 2–5-year-old children, Hispanic children in this sample have a prevalence of obesity that is 5% higher than all other racial or ethnic groups. National data [2] have found an even larger gap with an estimate of 15.6% (95% CI: 12.5–19.2) in Hispanic 2–5-year-olds and 5.2% (95% CI: 3.1–8.3) in white children; data from Massachusetts showed a similar gap to the national data with an estimate of 11.8–12.9% for Hispanic children less than 6 years old compared with 6.2–8.5% for white children [15].

The map data demonstrate the variation in obesity across San Antonio and suggest a decrease between 2009 and 2015 in areas of San Antonio with very high levels of obesity; however, the only decreases noted in the regression analysis were in the youngest children. The higher prevalence zip codes generally correspond to lower reported income zip codes in San Antonio [22]. These maps also highlight areas of significant disparity by ethnicity, highlighting the complex drivers of obesity with both environmental and cultural components.

A strength of this study is its sheer size with over 300,000 measurements representing over 100,000 children. A limitation is the nonrandom nature of the sample. While the health system does have clinics spread throughout San Antonio, it is also a major academic center with a large referral population which may select for a more morbid population, potentially with higher obesity rates than the population of children at large. Utilization of these local, nonrandom data in comparison with the few random samples available nationally, with appropriate limitations, can allow for a better understanding of obesity trends and variation. Another limitation is the lack of a specific measure of socioeconomic status which would have allowed for examination not only by race/ethnicity but accounting for disparities by socioeconomic status as well. Variations in obesity prevalence have also been observed across countries of origin for adults of Hispanic ethnicity [23], and our study did not have that information available to compare.

## Supplementary Material

Sample sizes for Estimates.

## Figures and Tables

**Table 1:** Prevalence of overweight (≥85th and <95th percentile) and obesity (≥95th percentile) by age group, race, and ethnicity, data from all years (2009–2015) combined.

	Age 2–17 years	Age 2–5 years	Age 6–11 years	Age 12–17 years
Overweight				
*All groups*	16.2 (16.0–16.5)	13.4 (13.1–13.8)	16.7 (16.3–17.0)	18.5 (18.1–18.9)
Male	15.2 (14.9–15.5)	13.4 (12.9–13.9)	15.5 (15.0–16.0)	16.5 (16.0–17.0)
Female	17.4 (17.1–17.7)	13.5 (13.0–14.0)	17.9 (17.3–18.4)	20.4 (19.9–21.0)
*Hispanic*	17.0 (16.7–17.2)	14.2 (13.8–14.6)	17.2 (16.7–17.6)	19.4 (19.0–19.8)
Male	15.7 (15.4–16.1)	14.1 (13.5–14.6)	15.9 (15.3–16.5)	17.2 (16.6–17.8)
Female	18.2 (17.9–18.6)	14.4 (13.8–14.9)	18.5 (17.8–19.1)	21.5 (20.9–22.1)
*White*	14.0 (13.5–14.6)	10.7 (9.8–11.6)	15.3 (14.3–16.4)	15.9 (14.9–16.9)
Male	13.3 (12.5–14.1)	11.0 (9.8–12.4)	14.4 (13.1–15.9)	14.3 (13.0–15.8)
Female	14.8 (14.0–15.7)	10.3 (9.1–11.6)	16.2 (14.8–17.8)	17.3 (15.9–18.8)
*Black*	14.3 (13.5–15.1)	11.7 (10.5–13.1)	15.3 (13.9–16.8)	15.8 (14.5–17.2)
Male	13.5 (12.5–14.7)	12.0 (10.3–13.9)	14.7 (12.8–16.8)	13.9 (12.2–15.9)
Female	15.1 (14.0–16.4)	11.4 (9.6–13.3)	16.0 (13.9–18.3)	17.6 (15.7–19.7)
*Asian*	9.9 (8.8–11.1)	7.9 (6.3–9.8)	12.4 (10.4–14.8)	9.4 (7.5–11.7)
Male	11.0 (9.4–12.9)	9.2 (6.9–12.2)	13.6 (10.6–17.4)	10.6 (7.8–14.2)
Female	8.8 (7.3–10.5)	6.4 (4.4–9.2)	11.4 (8.7–14.6)	8.4 (6.1–11.5)
Obese				
*All groups*	23.4 (23.2–23.7)	14.5 (14.2–14.9)	26.8 (26.4–27.3)	28.6 (28.1–29.0)
Male	24.8 (24.5–25.2)	15.1 (14.6–15.6)	29.1 (28.5–29.8)	30.3 (29.7–30.9)
Female	22.0 (21.6–22.3)	13.5 (13.0–14.0)	24.4 (23.8–25.1)	27.0 (26.4–27.6)
*Hispanic*	25.3 (25.1–25.6)	15.9 (15.5–16.4)	29.5 (29.0–30.0)	30.3 (29.8–30.8)
Male	27.1 (26.7–27.5)	16.6 (16.0–17.2)	32.3 (31.5–32.0)	32.4 (31.7–33.1)
Female	23.6 (23.2–24.0)	15.2 (14.6–15.8)	26.6 (25.9–27.3)	28.4 (27.7–29.1)
*White*	16.6 (16.0–17.2)	9.6 (8.7–10.5)	16.3 (15.3–17.4)	23.1 (22.0–24.3)
Male	17.5 (16.6–18.3)	9.8 (8.7–11.1)	17.5 (16.0–19.1)	24.7 (23.0–26.5)
Female	15.8 (14.9–16.6)	9.2 (8.1–10.5)	15.0 (13.6–16.6)	21.6 (20.1–23.2)
*Black*	18.2 (17.3–19.1)	8.9 (7.8–10.1)	19.8 (18.3–21.5)	24.9 (23.3–26.6)
Male	16.8 (15.6–18.0)	9.2 (7.7–10.9)	18.1 (16.0–20.3)	22.7 (20.5–25.0)
Female	19.6 (18.4–21.0)	8.6 (7.1–10.4)	21.9 (19.5–24.5)	27.1 (24.8–29.5)
*Asian*	8.5 (7.4–9.6)	7.2 (5.7–9.1)	11.9 (9.8–14.2)	6.2 (4.7–8.1)
Male	10.8 (9.2–12.7)	9.6 (7.3–12.6)	15.1 (12.0–19.0)	7.5 (5.2–10.7)
Female	6.2 (5.0–7.7)	4.5 (2.9–7.0)	8.9 (6.6–11.9)	5.0 (3.2–7.5)

**Table 2:** Regression results of year on obesity (dependent variable) accounting for repeated measures within individuals, with covariates of sex, race/ethnicity, and age group, presented as odds ratios (95% CI), *p* value, in San Antonio, Texas.

Variable	Estimate
*Year *	
2015	0.93 (0.88–0.97), 0.002
2014	0.91 (0.87–0.96), <0.001
2013	0.90 (0.85–0.95), <0.001
2012	0.91 (0.87–0.96), 0.004
2011	0.90 (0.85–0.94), <0.001
2010	0.95 (0.91–1.00), 0.03
2009	1.00 (reference)
*Gender *	
Male	1.00 (reference)
Female	0.84 (0.81–0.87), <0.001
*Race/ethnicity *	
White	1.00 (reference)
Hispanic	1.71 (1.61–1.82), <0.001
Black	1.09 (0.99–1.20), 0.08
Asian	0.44 (0.36–0.53), <0.001
*Age *	
2–5 years	1.00 (reference)
6–11 years	1.79 (1.73–1.86), <0.001
12–17 years	1.99 (1.92–2.07), <0.001

**Table 3:** Regression results of year on obesity (dependent variable) accounting for repeated measures within individuals and adjusted for sex, stratified by race/ethnicity, presented as odds ratios (95% CI), *p* value, in San Antonio, Texas. Odds ratios indicate the value for the independent variable of year of measurement with 2009 for that race/ethnicity and age stratum as the reference.

	All ages^1^	2–5 years	6–11 years	12–17 years
*Hispanic*				
2015	0.94 (0.89–0.99), 0.02	0.85 (0.75–0.97), 0.02	0.98 (0.90–1.06), 0.57	1.00 (0.92–1.09), 0.98
2014	0.92 (0.88–0.97), 0.003	0.84 (0.73–0.96), 0.009	0.93 (0.85–1.01), 0.10	1.00 (0.92–1.08), 0.94
2013	0.91 (0.87–0.97), 0.001	0.78 (0.68–0.90), 0.001	0.93 (0.85–1.01), 0.09	1.00 (0.92–1.09), 0.99
2012	0.94 (0.88–1.00), 0.04	0.81 (0.70–0.94), 0.005	1.02 (0.92–0.85), 0.76	0.97 (0.88–1.06), 0.50
2011	0.90 (0.85–0.96), <0.001	0.83 (0.72–0.95), 0.009	0.95 (0.87–1.04), 0.24	0.96 (0.88–1.05), 0.39
2010	0.94 (0.90–0.99), 0.03	0.91 (0.80–1.04), 0.17	0.98 (0.91–1.06), 0.60	0.97 (0.90–1.05), 0.45
Female (reference = male)	0.82 (0.79–0.85), <0.001	0.89 (0.82–0.95), 0.001	0.76 (0.71–0.81), <0.001	0.83 (0.79–0.88), <0.001
*White*				
2015	0.80 (0.68–0.94), 0.008	0.68 (0.47–1.00), 0.05	0.98 (0.75–1.28), 0.88	0.84 (0.66–1.06), 0.13
2014	0.80 (0.68–0.94), 0.006	0.69 (0.47–1.01), 0.06	0.97 (0.74–1.27), 0.83	0.82 (0.65–1.03), 0.09
2013	0.77 (0.65–0.92), 0.003	0.72 (0.48–1.09), 0.12	0.86 (0.65–1.15), 0.31	0.81 (0.64–1.02), 0.08
2012	0.81 (0.67–0.96), 0.02	0.66 (0.43–1.02), 0.06	0.78 (0.57–1.08), 0.13	0.89 (0.69–1.16), 0.39
2011	0.85 (0.72–1.01), 0.06	0.67 (0.43–1.03), 0.07	0.85 (0.65–1.12), 0.24	0.99 (0.77–1.27), 0.93
2010	0.95 (0.81–1.12), 0.054	0.87 (0.59–1.29), 0.49	0.96 (0.76–1.21), 0.74	1.02 (0.82–1.28), 0.83
Female (reference = male)	0.89 (0.79–0.99), 0.04	0.92 (0.73–1.15), 0.46	0.85 (0.70–1.03), 0.09	0.87 (0.74–1.02), 0.08
*Black*				
2015	0.92 (0.74–1.14), 0.45	0.61 (0.36–1.03), 0.07	0.88 (0.61–1.28), 0.51	1.30 (0.91–1.85), 0.15
2014	0.84 (0.67–1.04), 0.11	0.47 (0.28–0.82), 0.007	0.88 (0.61–1.27), 0.51	1.24 (0.88–1.75), 0.22
2013	0.79 (0.63–0.99), 0.04	0.55 (0.32–0.97), 0.04	0.82 (0.56–1.20), 0.30	1.02 (0.72–1.44), 0.93
2012	0.81 (0.64–1.03), 0.09	0.61 (0.34–1.08), 0.09	0.82 (0.54–1.24), 0.35	1.05 (0.72–1.54), 0.80
2011	0.84 (0.67–1.05), 0.13	0.65 (0.37–1.16), 0.15	0.78 (0.52–1.15), 0.21	1.31 (0.91–1.90), 0.15
2010	0.96 (0.79–1.17), 0.67	1.15 (0.73–1.82), 0.54	0.91 (0.73–1.29), 0.84	0.95 (0.68–1.34), 0.77
Female (reference = male)	1.12 (0.96–1.30), 0.16	0.85 (0.62–1.18), 0.33	1.13 (0.88–1.46), 0.34	1.30 (1.04–1.62), 0.02
*Asian*				
2015	1.07 (0.64–1.79), 0.80	1.47 (0.51–4.27), 0.48	0.90 (0.45–1.82), 0.77	0.91 (0.30–2.79), 0.87
2014	0.97 (0.57–1.65), 0.91	1.16 (0.37–3.58), 0.80	0.99 (0.50–1.97), 0.97	1.02 (0.31–3.37), 0.97
2013	1.23 (0.72–2.10), 0.45	2.24 (0.78–6.45), 0.13	0.90 (0.43–1.88), 0.77	1.15 (0.34–3.85), 0.82
2012	1.07 (0.61–1.87), 0.83	1.74 (0.53–5.70), 0.36	0.72 (0.34–1.54), 0.40	1.12 (0.33–3.83), 0.85
2011	0.75 (0.39–1.44), 0.39	1.68 (0.53–5.38), 0.38	0.29 (0.12–0.70), 0.006	1.02 (0.29–3.57), 0.98
2010	1.00 (0.56–1.80), 1.00	1.15 (0.37–3.59), 0.82	0.62 (0.26–1.50), 0.29	1.49 (0.47–4.75), 0.50
Female (reference = male)	0.59 (0.41–0.86), 0.005	0.47 (0.24–0.93), 0.03	0.58 (0.35–0.95), 0.03	0.82 (0.41–1.63), 0.57

^1^Multivariable generalized linear model with all age groups included but adjusted for age.

## References

[1] Ogden C. L., Carroll M. D., Kit B. K., Flegal K. M. (2014). Prevalence of childhood and adult obesity in the United States, 2011-2012. *The Journal of the American Medical Association*.

[2] Ogden C. L., Carroll M. D., Lawman H. G. (2016). Trends in obesity prevalence among children and adolescents in the United States, 1988–1994 through 2013-2014. *Journal of the American Medical Association*.

[3] Skinner A. C., Skelton J. A. (2014). Prevalence and trends in obesity among children in the United States, 1999–2012. *JAMA Pediatrics*.

[4] Singh A. S., Mulder C., Twisk J. W. R., van Mechelen W., Chinapaw M. J. M. (2008). Tracking of childhood overweight into adulthood: a systematic review of the literature. *Obesity Reviews*.

[5] Lazo M., Bilal U., Perez-Escamilla R. (2015). Epidemiology of NAFLD and Type 2 Diabetes: Health Disparities Among Persons of Hispanic Origin. *Current Diabetes Reports*.

[6] Koebnick C., Mohan Y. D., Li X., Young D. R. (2015). Secular Trends of Overweight and Obesity in Young Southern Californians 2008-2013. *The Journal of pediatrics*.

[7] Kharofa R. Y., Klein J. A., Khoury P., Siegel R. M. (2016). Severe Obesity Decreasing in Children in Cincinnati, Ohio. *Clinical Pediatrics*.

[8] Centers for Disease Control and Prevention (CDC). Obesity prevalence among low-income, preschool-aged children—New York City and Los Angeles County, 2003–2011. MMWR Morb Mortal Wkly Rep. 2013;62: 17–22 PMC4604837 23325351

[9] Weedn A. E., Hale J. J., Thompson D. M., Darden P. M. (2014). Trends in obesity prevalence and disparities among low-income children in Oklahoma, 2005-2010. *Childhood Obesity*.

[10] Obesity in Bexar County. In: City of San Antonio Metropolitan Health District [Internet]. 2013, https://www.sanantonio.gov/Portals/0/Files/health/HealthyLiving/FactSheet-Obesity.pdf

[11] Flegal K. M., Wei R., Ogden C. L., Freedman D. S., Johnson C. L., Curtin L. R. (2009). Characterizing extreme values of body mass index-for-age by using the 2000 Centers for Disease Control and Prevention growth charts. *The American Journal of Clinical Nutrition*.

[12] Levi J., Segal L., Rayburn J., Martin A. http://stateofobesity.org/files/stateofobesity2015.pdf.

[13] Pan L., Freedman D. S., Sharma A. J. (2016). Trends in Obesity Among Participants Aged 2–4 Years in the Special Supplemental Nutrition Program for Women, Infants, and Children—United States, 2000–2014. *MMWR. Morbidity and Mortality Weekly Report*.

[14] Li W., Buszkiewicz J. H., Leibowitz R. B., Gapinski M. A., Nasuti L. J., Land T. G. (2015). Declining trends and widening disparities in overweight and obesity prevalence among Massachusetts public school districts, 2009-2014. *American Journal of Public Health*.

[15] Wen X., Gillman M. W., Rifas-Shiman S. L., Sherry B., Kleinman K., Taveras E. M. (2012). Decreasing prevalence of obesity among young children in Massachusetts from 2004 to 2008. *Pediatrics*.

[16] Tilling K., Davies N. M., Nicoli E. (2011). Associations of growth trajectories in infancy and early childhood with later childhood outcomes. *American Journal of Clinical Nutrition*.

[17] Ekelund U., Ong K., Linné Y. (2006). Upward weight percentile crossing in infancy and early childhood independently predicts fat mass in young adults: the Stockholm Weight Development Study (SWEDES). *American Journal of Clinical Nutrition*.

[18] Lumeng J. C., Kaciroti N., Sturza J. (2015). Changes in body mass index associated with head start participation. *Pediatrics*.

[19] Hesketh K. D., Campbell K. J. (2010). Interventions to prevent obesity in 0-5 year olds: an updated systematic review of the literature. *Obesity*.

[20] Waters E., de Silva-Sanigorski A., Burford B. J. (2011). Interventions for preventing obesity in children. *Cochrane Database of Systematic Reviews*.

[21] Oude Luttikhuis H., Baur L., Jansen H. (2009). Interventions for treating obesity in children. *Cochrane Database of Systematic Reviews*.

[22] Internal Revenue Service [Internet]. 2014, https://www.irs.gov/uac/soi-tax-stats-individual-income-tax-statistics-zip-code-data-soi

[23] Daviglus M. L., Talavera G. A., Avilés-Santa M. L. (2012). Prevalence of major cardiovascular risk factors and cardiovascular diseases among Hispanic/Latino individuals of diverse backgrounds in the United States. *JAMA: Journal of the American Medical Association*.

