# Supplementary material for: Trends and Disparities in the Prevalence of Childhood Obesity in South Texas between 2009 and 2015
Source: J Obes. 2017 Jul 18;2017:1424968. doi: 10.1155/2017/1424968 (PMC5540384; doi:10.1155/2017/1424968)
Supplement: Supplementary file 1 — Sample sizes for Estimates. [file 1424968.f1.pdf]

Supplemental Table 1: Sample sizes for children and adolescents (age 2-17 years) included in the analysis, all children with biologically plausible BMI z-scores included, by race and ethnicity, stratified by age group

Age group	All	Hispanic	black	white	Asian
**All ages**	114,406	90,315	7,346	14,239	2,506
2-5	37,349	29,616	2,375	4,468	890
6-11	37,045	29,236	2,283	4,674	852
12-17	40,012	31,463	2,688	5,097	764
**Males**	57,578	45,307	3,788	7,252	1,231
2-5	19,104	15,129	1,240	2,357	15,129
6-11	18,976	14,972	1,227	2,439	14,972
12-17	19,343	15,206	1,321	2,456	15,206
**Females**	56,828	45,008	3,558	6,987	1,275
2-5	18,155	14,487	1,135	2,111	422
6-11	18,004	14,264	1,056	2,235	449
12-17	20,669	16,257	1,367	2,641	404
